# CLSI-based verification and *de novo* establishment of reference intervals for common biochemical assays in Croatian newborns

**DOI:** 10.11613/BM.2024.020705

**Published:** 2024-04-15

**Authors:** Iva Friščić, Sonja Perkov, Andrea Radeljak, Jasminka Stipanović-Kastelić, Mirjana Mariana Kardum Paro

**Affiliations:** 1 Department of Medical Biochemistry and Laboratory Medicine, University Hospital Merkur, Zagreb, Croatia; 2 Department of Neonatology with Intensive Care, University Hospital Merkur, Zagreb, Croatia

**Keywords:** reference values, reference range, neonatology, pediatrics, biochemistry

## Abstract

**Introduction:**

This study aimed to examine whether the Canadian Laboratory Initiative on Pediatric Reference Intervals (CALIPER) reference intervals for 19 commonly used biochemical assays (potassium, sodium, chloride, calcium, magnesium, inorganic phosphorous, glucose, urea, creatinine, direct and total bilirubin, C-reactive protein (CRP), total protein, albumin, aspartate aminotransferase (AST), alanine aminotransferase (ALT), gamma-glutamyl transferase (GGT), alkaline phosphatase (ALP) and lactate dehydrogenase (LD)) could be applied to the newborn population of one Croatian clinical hospital.

**Materials and methods:**

Reference interval verification was performed according to the CLSI EP28-A3c guidelines. Samples of healthy newborns were selected using the direct *a posteriori* sampling method and analyzed on the Beckman Coulter AU680 biochemical analyzer. If verification wasn’t satisfactory, further procedure included *de novo* determination of own reference intervals by analyzing 120 samples of healthy newborns.

**Results:**

After the first set of measurements, 14/19 tested reference intervals were adopted for use: calcium, inorganic phosphorous, glucose, urea, creatinine, total bilirubin, CRP, total protein, albumin, AST, ALT, GGT, ALP and LD. A second set of samples was tested for 5 analytes: potassium, sodium, chloride, magnesium and direct bilirubin. The verification results of the additional samples for sodium and chloride were satisfactory, while the results for potassium, magnesium and direct bilirubin remained unsatisfactory and new reference intervals were determined.

**Conclusions:**

The CALIPER reference intervals can be implemented into routine laboratory and clinical practice for the tested newborn population for most of the analyzed assays, while own reference intervals for potassium, magnesium and direct bilirubin have been determined.

## Graphical abstract

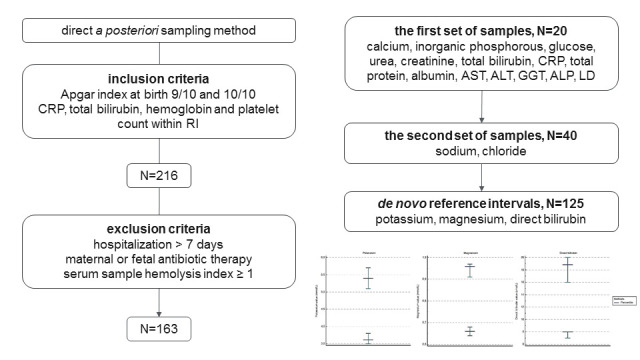

## Highlights

• Nineteen most commonly used biochemical assays on Beckman Coulter AU680

• Harmonized reference intervals are suitable for most assays

*• De novo* reference intervals for potassium, magnesium and direct bilirubin were determined

## Introduction

Reliable and accurate reference intervals are a fundamental tool for an appropriate interpretation of laboratory test results and thus have a significant effect on the clinical decision-making process (*1*). Currently, there are still many challenges in verification and establishment of reference intervals, especially for the neonatal and pediatric population, resulting in the inappropriate interpretation of many pediatric laboratory test results due to improper reference intervals, generally derived from adult populations, hospitalized pediatric populations or from outdated technology (*1*-*3*). The most demanding part in the verification and establishment of pediatric reference intervals includes the collection of a sufficient number of samples from healthy, referent persons, mainly due to ethical restrictions regarding venipuncture of children and newborns without clinical indication (*1*). A possible solution to this obstacle includes the analysis of residual samples after routine testing and the following selection of healthy individuals using the direct *a posteriori* sampling method (*3*, *4*). The international guideline of the Clinical and Laboratory Standards Institute (CLSI), CLSI 28-A3c, which is the most widely used reference in this area, states that at least 120 samples are required to determine the 95th percentile reference limit with a 90% confidence interval (*4*, *5*). Since children of different gender and age differ greatly in physical, immune and hormonal characteristics, it is necessary to form partitions of reference intervals, *i.e.* separate reference intervals by gender and age groups as well as separate reference intervals for newborns and premature infants (*3*, *4*).

According to the International Organization for Standardization (ISO) standard 15189, ensuring accurate reference intervals and clinically actionable cutoff values that provide accurate context for result interpretation is the obligation of every individual laboratory (*1*, *2*, *6*). *De novo* establishment of reference intervals is for most individual laboratories practically impossible, as it is too time- and cost-consuming for routine practice (*6*, *7*). Possible solutions for this problem include transference and verification of reference intervals from various sources, including manufacturers’ test instructions, national or international expert group publications or harmonized reference intervals determined by direct single- or multicenter studies. Harmonized reference interval studies have a well-defined reference population, optimal control of preanalytical and analytical variables and narrow confidence limits around the established reference intervals (*1*, *2*, *6*, *7*). In Croatia, harmonized reference intervals are used for most analytes, but the last revision of the used reference intervals by age group was carried out in 2016, which is why it was necessary to review recent literature data on studies of harmonized reference intervals (*8*). Several national and international initiatives for pediatric reference interval harmonization have been formed during the past two decades, the most comprehensive study being the Canadian Laboratory Initiative on Pediatric Reference Intervals (CALIPER) (*3*, *4*, *7*). In this study, most of the reference intervals were initially established on Abbott assays, but since then transference of CALIPER reference intervals has been performed on various other manufacturers’ platforms (*4*, *7*, *9*-*13*). The transference was performed according to the international guidelines CLSI 28-A3c (*5*). One of the other analytical systems to which the CALIPER reference intervals were transferred was the Beckman Coulter AU biochemical analyzer (Beckman Coulter, Brea, USA), the same analytical system used in our laboratory (*9*-*11*). Consequently, it was our goal to perform the transference and verification of the CALIPER reference intervals of the most commonly used biochemical analytes required for our neonatal population from the Department of Neonatology with Intensive Care at Merkur University Hospital.

## Materials and methods

### Subjects

This study was approved by the Ethical Committee of Merkur University Hospital, Zagreb, Croatia (approval number: 03/1-4700). It was performed between March and October 2022. Referent persons were selected using the direct *a posteriori* sampling method, among newborns younger than 15 days whose blood was sampled for routine sample analysis. The newborns that were included in this study had an Apgar score at birth of at least 9/10 and C-reactive protein (CRP) and total bilirubin concentrations within the reference intervals of both CALIPER reference intervals and the reference intervals recommended by Beckman Coulter, the manufacturer of reagents used in this study. After sample analysis, further exclusion criteria included hospitalization lasting longer than 7 days, maternal or newborn antibiotic therapy, hemoglobin and platelet count below the lower limit of their reference intervals for Sysmex XN-3000 analyzers from the CALIPER database and hemolysis index ≥ 1.

### Methods

Routine blood sampling protocol at the Department of Neonatology with Intensive Care at Merkur University Hospital includes blood sampling of newborns with a clinical indication that includes fever of mother or newborn, jaundice, low or high birth weight, preterm delivery, protracted labor, other clinical indications assessed by the physician and monitoring of these conditions. Nurses draw capillary blood from the index or middle finger of the newborns into BD microtainers (Becton, Dickinson and Company, USA). Blood for hematological analysis is sampled to BD K2EDTA microtainers with the volume of 250-500 µL and blood for biochemical analysis is sampled to BD Clot activator/SST gel microtainers with a volume of 400-600 µL. Samples are delivered to the laboratory as a matter of priority, within 15 minutes from sampling.

Laboratory personnel received the samples, rejecting samples with volumes < 250 µL (hematological analysis) and < 400 µL (biochemical analysis) due to insufficient sample volume. Hematological analysis was carried out immediately on the Sysmex XN1000 analyzer (Sysmex, Kobe, Japan). BD Clot activator/SST gel microtainers for biochemical analysis were centrifuged for 10 minutes at 1300-2000xg at room temperature (18-25 °C) to obtain serum samples. After centrifugation, serum samples were analyzed on the Beckman Coulter AU680 biochemical analyzer (Beckman Coulter, Brea, USA). During the study, for every newborn who had sampled both hematological and biochemical test tubes, in addition to the tests ordered by the physician, there were simultaneously determined HIL indices, potassium, sodium, chloride, total bilirubin and CRP. Samples with hemolysis index ≥ 1 were automatically excluded from the study. If other analytical exclusion criteria were satisfied (hemoglobin and platelet count, CRP and total bilirubin within reference intervals), further analysis for other parameters for which reference intervals verification was performed followed. Due to the small volume of neonatal serum samples, it was not always possible to evaluate the samples across all parameters, rather, a partial analysis for some samples was performed. All analysis was completed during the turnaround time (TAT) for newborn samples at our laboratory, which is 1 hour from sample admission.

Reference interval verification was performed for 19 analytes on the Beckman Coulter AU680 biochemical analyzer, using Beckman Coulter reagents. The following 19 analytes were measured: potassium, sodium, chloride (indirect potentiometry), calcium (photometric test, o-cresolphthalein-complexone), magnesium (photometric test, xylidyl blue), inorganic phosphorous (photometric UV test, molibdate), glucose (hexokinase method), creatinine (enzymatic), direct (3,5-dichlorophenyldiazonium tetrafluoroborate, DPD) and total bilirubin (DPD with caffeine), CRP (immunoturbidimetry), total protein, albumin (photometric test), urea, aspartate aminotransferase (AST), alanine aminotransferase (ALT), lactate dehydrogenase (LD) (kinetic UV test), gamma-glutamyl transferase (GGT) and alkaline phosphatase (ALP) (kinetic color test). HIL indices were determined with the Beckman Coulter photometric test and assessed semi-quantitative, by the approximate concentration of the interferent.

### Statistical analysis

All obtained data are presented in a table, and the first set of samples that met the exclusion criteria was always used for the verification. According to the international CLSI 28-A3c guidelines, verification of the reference intervals is performed by analyzing 20 samples (*3*). Considering that the reference intervals for all the listed analytes for newborns younger than 15 days are gender-independent, the analysis was not performed for both genders, but on a total of 20 samples (*3*, *4*). A reference interval was adopted if at least 18/20 results were within the CALIPER reference intervals, including 95% confidence intervals around the upper and lower limits of each reference interval. For analytes for which this criterion was not met in the first set of samples, a new set of additional 20 samples was analyzed. If the criterion still wasn’t met, the further procedure included the determination of own reference intervals by analyzing a minimum of 120 samples of healthy newborns (*5*).

Outlier exclusion was performed by visual inspection of the box and whisker plots and the method of outlier exclusion by Tukey (1977). All outliers were excluded from the study after which the data was tested for additional outliers. The normality of the sample data distribution was tested using the Kolmogorov-Smirnov test with a statistically significant P < 0.05. The data was transformed so it would follow a normal distribution using the Box-Cox transformation using the λ value of 1. Finally, the reference intervals were calculated as double-sided reference intervals by a method based on a normal distribution, which was possible because of the data transformation. The reference intervals included 95% of the population from which the reference subjects were chosen and the lower and upper reference limits were estimated as the 2,5th and 97,5th percentiles of the distribution for the reference population. Due to the inclusion of more than 120 results in the calculation, it was possible to calculate 90% confidence intervals around the upper and lower reference limits for each analyte. All statistical analyses were performed using MedCalc for Windows, version 17.9.2 (MedCalc Software, Ostend, Belgium).

## Results

After applying exclusion criteria, 163 samples were collected, whereby 76 samples (47%) belonged to female and 87 samples (53%) to male newborns. The median age of the newborns was 3 days, ranging from 2 to 11 days.

**Figure 1:**
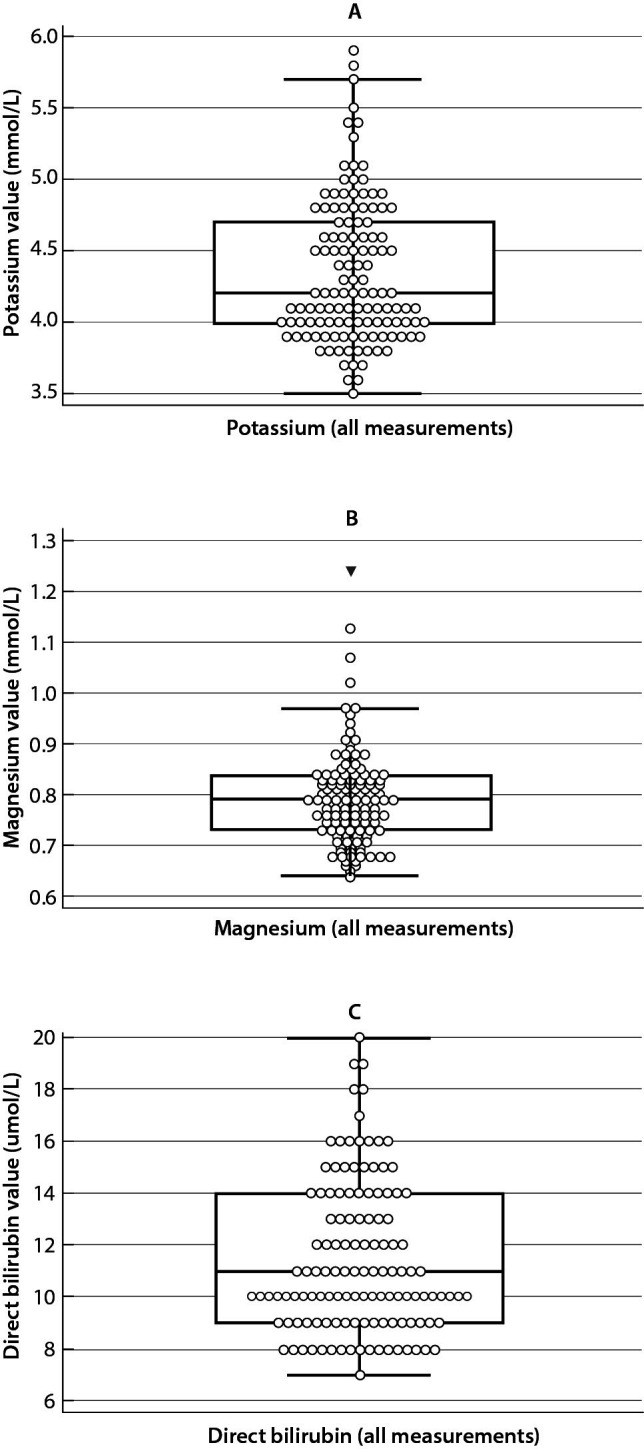
Box and whisker plots for potassium (A), magnesium (B) and direct bilirubin (C) before outlier exclusion. The plots show the distribution of the data. The dots outside the minimum and maximum bars are outliers and the bolded triangle in the box and whisker plot for magnesium (B) is a far-out value. There were two outliers for potassium, four for magnesium and none for direct bilirubin

**Figure 2:**
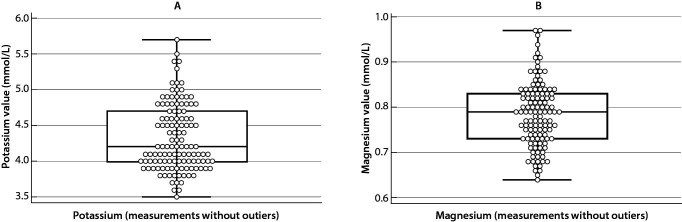
Box and whisker plots for potassium (A) and magnesium (B) after outlier exclusion. The plots show the distribution of the data. No outliers were found.

Using the outlier exclusion method, two outliers were found for potassium, four for magnesium and none for direct bilirubin (Figure 1). Once the outliers had been eliminated, no more outliers had been not found (Figure 2). By testing the normality of the sample data distribution, a non-Gaussian distribution for all three analytes was obtained (P < 0.001 for all three analytes).

After the first set of measurements, 14 of total 19 tested reference intervals were adopted for use: calcium, inorganic phosphorous, glucose, urea, creatinine, total bilirubin, CRP, total protein, albumin, AST, ALT, GGT, ALP and LD. A second set of 20 samples was tested for 5 remaining analytes: potassium, sodium, chloride, magnesium and direct bilirubin. The results of the additional samples for sodium and chloride were within the examined reference intervals, while the results for potassium, magnesium and direct bilirubin remained unsatisfactory. Verification results for all analytes, including sample size, median, interquartile range (IQR) and number of samples within reference interval are summarized in Table 1.

**Table 1:** CALIPER reference interval verification results for all analytes

**Analyte**	**Sample size**	**Median (IQR), first set of samples**	**Median (IQR), second set of samples**	**CALIPER reference interval**	**Samples within the CALIPER reference interval (first set of samples, N)**	**Samples within the CALIPER reference interval (second set of samples, N)**
Potassium, mmol/L	40	4.1 (4.0 - 4.5)	4.1 (3.9 - 4.6)	4.2 - 6.2	13/20	12/20
Sodium, mmol/L	40	140 (139 - 142)	140 (139 - 143)	139 - 146	17/20	18/20
Chloride, mmol/L	40	107 (105 - 109)	107 (106 - 108)	104 - 109	17/20	18/20
Calcium, mmol/L	20	2.43 (2.35 - 2.51)	/	2.17 - 2.74	19/20	/
Magnesium, mmol/L	40	0.79 (0.75 - 0.83)	0.78 (0.74 - 0.83)	0.79 - 1.56	14/20	15/20
Phosphorous, mmol/L	20	2.15 (1.92 - 2.31)	/	1.76 - 3.37	19/20	/
Glucose, mmol/L	20	4.5 (4.3 - 5.0)	/	4.0 - 6.2	19/20	/
Urea, mmol/L	20	3.1 (2.6 - 3.8)	/	1.1 - 8.0	20/20	/
Creatinine, µmol/L	20	47 (39 - 61)		25 - 73	20/20	/
Total bilirubin, µmol/L	20	174 (137 - 188)	/	4 - 253	20/20	/
Direct bilirubin, µmol/L	40	11 (10 - 12)	10 (9 - 13)	3 - 8	4/20	4/20
CRP, mg/L	20	1.8 (0.9 - 3.6)	/	0.3 - 5.8	20/20	/
Total protein, g/L	20	58 (54 - 61)	/	52 - 79	20/20	/
Albumin, g/L	20	36.5 (35.4 - 38.7)	/	31 - 43	20/20	/
AST, U/L	20	47 (38 - 68)		30 - 146	20/20	
ALT, U/L	20	21 (14 - 22)	/	6 - 30	18/20	/
GGT, U/L	20	100 (76 - 132)	/	17 - 158	19/20	/
ALP, U/L	20	156 (128 - 179)	/	76 - 233	20/20	/
LD, U/L	20	544 (468 - 605)	/	256 - 1017	20/20	/
CALIPER - Canadian Laboratory Initiative on Pediatric Reference Intervals. IQR - interquartile range. CRP - C-reactive protein. AST - aspartate aminotransferase. ALT - alanine aminotransferase. GGT - gamma-glutamyl transferase. ALP - alkaline phosphatase. LD - lactate dehydrogenase.						

Considering that the verification results for potassium, magnesium and direct bilirubin were not satisfactory, *de novo* reference intervals were determined. A total of 125 samples were collected and analyzed for each analyte. The results are shown in Table 2.

**Table 2:** *De novo* reference intervals for potassium, magnesium and direct bilirubin

**Analyte**	**Sample size (outliers excluded)**	**Median (IQR)**	**Reference interval** **95th percentile**	**Lower limit** **90% CI**	**Upper limit** **90% CI**
Potassium, mmol/L	123	4.2 (4.0 - 4.7)	3.4 - 5.2	3.3 - 3.5	5.1 - 5.3
Magnesium, mmol/L	121	0.79 (0.73 - 0.83)	0.65 - 0.92	0.63 - 0.66	0.90 - 0.94
Direct bilirubin, µmol/L	125	11 (9 - 14)	6 - 17	5 - 6	16 - 18
CI - confidence interval. IQR - interquartile range.					

## Discussion

The results of this study showed that the CALIPER reference intervals for sodium, chloride, calcium, inorganic phosphorous, glucose, urea, creatinine, total bilirubin, CRP, total protein, albumin, AST, ALT, GGT, ALP and LD can be implemented into routine laboratory and clinical practice for the newborn population of the Department of Neonatology with Intensive Care at Merkur University Hospital. Those criteria are not met for potassium, magnesium and direct bilirubin. Reasons why one laboratory reference interval does not apply to another are the differences in the reference population or the analytical methods. Differences in the reference population may include environmental and geographic factors and ethnic differences, while differences in analytical methods include different measurement principles, calibration or reagent formulation (*3*).

All tested newborn samples originate from an ethnically homogenous Caucasian population from the Zagreb area, while the CALIPER cohort was composed of a multiethnic population. During the initial project, preliminary research on differences between individual ethnic groups was conducted where ethnic differences in seven biochemical analytes, including magnesium were shown (*14*). These findings resulted in a comprehensive Canadian pediatric study that examined ethnicity-specific biomarkers. Results showed ethnic-specific differences among seven biomarkers for which partitioned reference intervals were made, but none of these analytes included potassium, magnesium or direct bilirubin, for which no clinically significant difference between different ethnicities was found (*15*). Therefore, the difference in the composition of the population is not an explanation for the difference in determined reference intervals. Furthermore, differences between CALIPER and our own reference intervals can originate from the fact that CALIPER reference intervals were determined on a much larger population and that they used a direct sampling approach between healthy individuals, while we, due to ethical reasons, used residual samples from newborns that had a clinical indication for blood sampling (*4*).

Regarding the analytical methods, the same methods were used for magnesium and direct bilirubin as in the study in which the reference intervals were transferred from the Abbott analyzer to the Beckman Coulter AU analyzer (*9*). In this study, transference for potassium was not made, so our verification included a reference interval obtained from the Siemens ADVIA XPT/1800 analyzer set as the default analyzer on the CALIPER website (*16*). The method on both analyzers is indirect potentiometry and therefore the analyzers should not have significant methodological or analytical differences from each other. Despite all stated above, there were found statistically significant differences between populations and/or methods in the CALIPER reference intervals and our patient population and the need for *de novo* determination of three reference intervals for potassium, magnesium and direct bilirubin has appeared. The determined reference intervals are population- and analyzer-specific and are suitable for our specific population. Furthermore, the sampling technique used in this study was identical to the one for routine sample collection at the Department of Neonatology with Intensive Care, mimicking thereby everyday sampling conditions and quality of the samples which ensures clinical applicability of the verified and determined reference intervals.

In conclusion, the new reference intervals that are now used to assess the results for the neonatal population at the Department of Neonatology with Intensive Care at Merkur University Hospital are a combination of verified transferred CALIPER reference intervals for most of the analytes and *de novo* determined reference intervals for potassium, magnesium and direct bilirubin that are specifically based on healthy newborns from an ethnically homogenous Caucasian population from the Zagreb area.

## Data Availability

The data generated and analyzed in the presented study are available from the corresponding author on request.
